# The pre-med ambition: is it worth it?

**Published:** 2017-06-30

**Authors:** Ishan Aditya, Zhen Jason Fan

**Affiliations:** 1 McMaster University, Ontario, Canada

Presentations, research work, GPA, MCAT, extra curriculars, and volunteering. You could call it the “pre-med six-pack,” which, like a well-defined *rectus abdominis*, represent the “core” of an aspiring young physician. However, unlike one’s abdominal muscles, which experience fatigue after being put through a strenuous workout, these six core components are scheduled for a daily workout in the calendars of pre-medical students across the nation. There is no day off.

There are also no immediate rewards - no gold stars or pats on the back. Instead, we rely on the hope that if we work hard today, our dreams will someday come to fruition. But, there’s a lingering fear in the back of every aspiring med student’s mind: what if, our dream to become physicians isn’t as glorified as we convince ourselves that it is? What if, we are the 50% of physicians or residents who burn out and struggle to cope with the stress?^1^,^2^ Indeed, we expect the magnitude of responsibility and challenge we face today will only be trivial in the years to come. How will we be ready to face unforeseen hardships that may force us to completely question our desire to enter this profession?

The role of a physician is heavily glorified in most cultures and in today’s western society you can find the job of a physician near the top of the list of dream jobs. In North America, being a physician gives an individual special status and immediate respect as well as a great deal of material wealth. However, the reality is that physicians are not always held on a pedestal. We have heard stories of family physicians in Canada receiving abuse from their patients. In a national survey, two in five surveyed Canadian GPs stated they were subjected to some degree of abuse during their practice, with patients – the very people we desire to help – being the greatest perpetrators.^3^ We are certainly cognizant of the discourse between provincial governments and practicing physicians. It’s not hard for our bubble to be burst by the reality of what Canadian physicians face. We ask ourselves a serious question - is our struggle to go down this path actually worth it?

We think so. It is far too easy for us to enjoy the privilege of receiving a great education, among other luxuries, while many others do not benefit from the same opportunities. We lament about the stress that we face but we sometimes forget that the hardships we face along this path are incomparable to the struggle our future patients may endure. We want to be physicians who devote ourselves to empower and advocate for the communities that have helped build us into who we are today. We have already taken the initial impetus towards immersing ourselves in the clinical environment. From our experience with volunteering in emergency departments, operating rooms, and conducting clinical research, we have had a small taste of the genuinely heart-warming feeling of improving the lives of patients with whom we have interacted. The feeling of instilling confidence in a sick child to fight their illness, and even bringing a smile to their face, is absolutely surreal. Even from personal experience of being patients ourselves, we remember the times when a physician went out of their way to help us, and want to be in the position to do the same.

If we take a step back, our journey towards becoming physicians has helped us grow into talented, diligent, and genuine individuals. We can communicate, inquire, and think critically all while embracing a passion for lifelong learning. There is no doubt that we will experience frustration, fatigue, and failure. To us, this is all part of the pursuit towards a meaningful career in medicine. Should we ever receive the privilege, bearing a white coat becomes not only a symbol of care but represents the conviction to embark on a tireless journey to ultimately better the greater community.
